# The Impact of Enhancing Diet Quality or Dietary Supplementation of Flavor and Multi-Enzymes on Primiparous Lactating Sows

**DOI:** 10.3390/ani12121493

**Published:** 2022-06-08

**Authors:** Li Zhe, Rui Zhou, Peter Kappel Theil, Uffe Krogh, Lunxiang Yang, Yong Zhuo, Yan Lin, Shengyu Xu, Xuemei Jiang, Lingjie Huang, Lianqiang Che, Bin Feng, De Wu, Zhengfeng Fang

**Affiliations:** 1 Key Laboratory for Animal Disease-Resistance Nutrition of China Ministry of Education, Animal Nutrition Institute, Sichuan Agricultural University, 211 Huimin Road, Wenjiang District, Chengdu 611130, China; zhelimy@126.com (L.Z.); rui1zhou@163.com (R.Z.); ylunxiang@163.com (L.Y.); zhuoyong@sicau.edu.cn (Y.Z.); linyan936@163.com (Y.L.); shengyu_x@hotmail.com (S.X.); jiangleehom@163.com (X.J.); 71424@sicau.edu.cn (L.H.); clianqiang@hotmail.com (L.C.); fengbin@sicau.edu.cn (B.F.); wude@sicau.edu.cn (D.W.); 2 Department of Animal Science, Aarhus University, Foulum, DK-8830 Tjele, Denmark; peter.theil@anis.au.dk; 3 SEGES Innovation Agro Food Park 15, DK-8200 Aarhus N, Denmark; uffekrogh@hotmail.com

**Keywords:** energy and nutrient density, flavor, multi-enzyme, primiparous lactating sow

## Abstract

**Simple Summary:**

Insufficient energy and nutrient ingestion of primiparous lactating sows causes excess body weight loss and oxidative stress and compromises piglet growth. Two strategies to enhance the daily intake of dietary energy and nutrients were compared with a standard lactation diet to understand the potential modes of action. We found that feeding on either high-quality or flavor plus multi-enzyme diets both improved sow feed digestibility and consequently increased the growth of piglets. In addition, the flavor plus multi-enzyme diet also improved the antioxidant capacity and health of sows. The data suggest that dietary supplementation with flavor and multi-enzymes may be more promising than a high-quality diet from a health and economic perspective like enhancing utilization of cereal byproducts and thus reducing expenditure of corn and soybeans.

**Abstract:**

This study was aimed to explore how a high-quality diet or a flavor plus multi-enzyme diet affects the feed intake, nutrient digestibility and antioxidation capacity of lactating sows and the growth of their progeny. Thirty primiparous sows were randomly assigned to three treatments from d 2 of lactation until weaning (d 21): control (CON), with a basal diet; high quality (HQ), with 200 kcal/kg higher net energy than CON; or the CON diet supplemented with 500 mg/kg flavor and 100 mg/kg multi-enzymes (F + E). Sows fed with the HQ or F + E diets improved piglets’ live weight (*p* < 0.05) and average daily weight gain (*p* < 0.10), litter weight gain (*p* < 0.10) and piglet growth to milk yield ratio (*p* < 0.10). Compared with CON, the HQ and F + E groups increased the digestibility of ether extract, ash, neutral detergent fiber, crude fiber and phosphorus (*p* < 0.10), and the HQ group also increased dry matter, gross energy, crude protein, acid detergent fiber and energy intake (*p* < 0.05). Compared with CON, the F + E group decreased serum urea nitrogen and aspartate aminotransferase (*p* < 0.05) and enhanced superoxide dismutase, catalase and glutathione peroxidase, but it decreased malondialdehyde in milk supernatant (*p* < 0.05).

## 1. Introduction

Previous studies reported that primiparous lactating sows generally have lower feed intake than multiparous sows, which results in insufficient energy and nutrient ingestion [1,2], causes excess body weight loss and oxidative stress and compromises the weight gain of their progeny [1,3]. Hence, strategies to increase the intake of energy, such as greater energy concentration in their diet or improved feed intake and digestibility through the addition of flavor and multi-enzymes, should be beneficial for sow performance, including reduced oxidative stress and enhanced piglet growth.

Flavor is widely used in diets for piglets and weaners [4,5] to improve feed intake and to ameliorate reduced feed intake post-weaning. The inclusion of flavor in sows’ diets has been shown to increase maternal feed intake and benefit the growth of their offspring [6,7,8,9]. Fiber content differs substantially across cereals, such as corn, wheat and barley, and they contain up to 9.0%, 11.3% and 18.6% non-starch polysaccharides (NSPs), respectively [10]. Unfortunately, pigs lack enzymes that are capable of digesting dietary NSPs [11]. The existence of NSPs may increase their water-holding capacity and digesta viscosity, which can increase the feed transit time and reduce the contact surface between enzymes and nutrients [12]. As a result, insufficient gastrointestinal capacity may reduce voluntary feed intake and nutrient digestibility [10,13,14,15]. A large number of references reported that the inclusion of NSP enzymes in livestock diet was shown to provide more energy and amino acids for animals [16,17,18]. Dietary supplementation with NSP enzymes can break down fibers into smaller fragments, which are further used by bacteria as a prebiotic, thus improving animal health and feed efficiency [19]. Therefore, it may be useful to combine NSP enzymes with increased palatability in the diet for lactating sows to improve the ingestion and digestion of energy and nutrients. 

In the present study, we hypothesized that higher dietary energy and nutrient concentrations due to the inclusion of oil and highly digestible feedstuffs benefits sows and suckling piglets by improving the daily intake of digestible nutrients and energy, and the dietary inclusion of flavor and multi-enzymes (NSPs plus phytase enzymes) also benefits sows and their offspring by improving both nutrient digestibility and feed intake. The objective of this study was to comparatively investigate the potential beneficial effects of enhanced dietary energy and nutrient concentrations or the dietary inclusion of flavor plus multi-enzymes in lactation diets on the performance and health of sows, as evaluated by the feed intake, nutrient digestibility, serum metabolite profile and antioxidant capacity of the sows and by the growth of suckling piglets.

## 2. Materials and Methods

All animal procedures in this experiment were approved by the Animal Care and Use Committee of Sichuan Agricultural University (Ya’an, China; Ethic Approval Code: SICAUAC201710-7). 

### 2.1. Experimental Animals and Design

A total of 30 primiparous sows (Duroc × (Landrace × Yorkshire)) with homogenous genetic backgrounds were randomly assigned to 3 dietary treatments on d 2 of lactation, and each treatment contained 10 replicates. The sows used in this experiment were selected from the same batch of weaning piglets and were reared to lactation on our research farm. Dietary treatments included: (1) control (CON), fed with a standard lactation diet; (2) high quality (HQ), a diet with 200 kcal/kg greater net energy concentration than CON, and the contents of crude protein, standardized ileal digestible amino acids, calcium and phosphorus were balanced relative to net energy as in CON; and (3) flavor plus multi-enzymes (F + E), a diet with 500 mg/kg flavor (Krave, Adisseo, Beijing, China) and 100 mg/kg multi-enzymes (Rovabio, Adisseo, Paris, France) on top of the CON diet. The main contents of flavor were benzyl acetate, γ-undecalactone and glucose. The multi-enzymes contained activities of xylanase, β-glucanase and phytase, which provide a minimum of 1250 U of xylanase, 860 U of β-glucanase and 1000 FTU of phytase per kg of diet. The experiment lasted from d 2 of lactation until weaning (d 21 of lactation), and the litter size was standardized to 9 pigs with similar litter weight (16.43 ± 0.13 kg) by cross-fostering on d 2 of lactation.

### 2.2. Experimental Diets and Husbandry

The HQ diet was formulated with highly digestible feedstuff according to the recommendation of the National Research Council 2012 for lactating sows [20]. Based on the results of a previous study about the multi-enzymes used in this study, the net energy of the CON and F + E groups was reduced by 200 kcal/kg compared with the HQ group. The fermented soybean meal was provided by Shanghai Yuanyao Biological Co. Ltd. (Shanghai, China), and the other ingredients were bought from the commercial market (Meishan, Sichuan, China). All the ingredients were bought from the same batch. The dietary ingredients and chemical compositions are shown in Table 1, and all diets were fed in a powder form. The sows were fed 3 kg on d 2 of lactation, and the feed amount increased by 1 kg per day from d 2 to 7 of lactation if the sows were able to eat up. Then, the sows were semi-ad libitum fed until weaning to reduce the residual in the trough between feedings. In other words, the feed was offered up to the amount described only when that feed was consumed by the sows. All the sows were fed three times daily. During the experimental period, no creep feed was used for piglets, and the sows and piglets had ad libitum access to water.

### 2.3. Sample Collection and Measurement

#### 2.3.1. Performance of Sows

The individual live weight and backfat thickness (BFT) were recorded after overnight fasting on d 2 and 21 of lactation for each sow. Backfat thickness was measured at the last rib, which was located 65 mm from the midline, using B-mode ultrasonography (Renco Lean-Meater 89372, Golden Valley, MN, USA). Predictions of the body lipids, body protein and body energy content of sows were conducted according to the method reported by Dourmad et al. (2008) [21], as follows:

Body fat (kg) = −26.4 + 0.221 × 0.96 × live weight (kg) + 1.331 × BFT (mm)

Body protein (kg) = 2.28 + 0.178 × 0.96 × live weight (kg) − 0.333 × BFT (mm)

Body energy (MJ) = −1074 + 13.65 × 0.96 × live weight (kg) + 45.94 × BFT (mm)

The changes in body composition were calculated based on the initial and final content of body fat, protein and energy of each individual sow. The feed consumption of sows was recorded weekly.

#### 2.3.2. Performance of Piglets and Predicted Milk Yield

The live weights of piglets were individually recorded on d 2, 7, 14 and 21 of lactation, and the body weights of dead piglets were recorded immediately and were included in the calculation of litter weight gain. The milk yield of sows was estimated based on daily litter weight gain and litter size according to the method described by Hansen et al. (2012) [22]. The piglet growth to milk yield ratio was also calculated based on weekly litter weight gain and weekly milk yield.

#### 2.3.3. Blood Sample Collection and Measurement

The blood samples of sows were collected from the ear vein after overnight fasting on d 7, 14 and 21 of lactation. The serum was collected after centrifuging at 1300× *g* for 15 min and was then stored at –20 °C until utilization. Serum creatinine (CREA), urea nitrogen (UN), β-hydroxybutyric acid (β-HBA), triglyceride (TG), non-esterified fatty acids (NEFA), low-density lipoprotein cholesterol (LDL-C), high-density lipoprotein cholesterol (HDL-C), total cholesterol (TC), aspartate aminotransferase (AST), alanine aminotransferase (ALT), gamma-glutamyl transferase (GGT) and C-reactive protein (CRP) were measured at the Institute of Animal Nutrition, Sichuan Agricultural University by using a fully automatic biochemical analyzer (HITACHI 3100, Tokyo, Japan) according to the instructions of kits (Nanjing Jiancheng Bioengineering Institute, Nanjing, China).

Furthermore, the activities of superoxide dismutase (SOD), catalase (CAT) and glutathione peroxidase (GSH-Px) and the concentrations of total antioxidant capacity (T-AOC) and malondialdehyde (MDA) in sow serum were measured following the instructions of kits (Nanjing Jiancheng Bioengineering Institute, Nanjing, China).

#### 2.3.4. Milk Sample Collection and Measurement

Milk samples were collected after ear vein injection with 2 mL of oxytocin diluent and 1 mL of oxytocin diluted by 1 mL normal saline on the morning of d 7, 14 and 21 after wiping the 2nd to 5th udder with ethyl alcohol. All the milk samples were collected in duplicate and were stored at –20 °C until further analysis. The milk samples were used to measure dry matter (DM), fat, protein, non-fat milk solids, lactose and UN by using an automatic analyzer (Foss MilkoScan FT+, Foss, Denmark) and somatic cell count (SCC) by using an SCC automatic analyzer (Foss Matic FC, Foss, Denmark) in New Hope Hongya Dairy Testing Center (Meishan, Sichuan, China). 

In addition, the milk supernatant was isolated by centrifuging at 8000× *g* for 15 min under 4 °C and was stored at −20 °C until analysis. The activities of SOD, CAT and GSH-Px and the contents of T-AOC and MDA in milk supernatant were measured following the guide of kits (Nanjing Jiancheng Bioengineering Institute, Nanjing, China).

#### 2.3.5. Apparent Total Tract Digestibility Sampling and Measurement

Fecal grab samples were collected from the rectum of each sow from d 18 to 21 of lactation. The fecal samples were mixed with 10 mL of 10% hydrochloric acid and three drips of methylbenzene per 100 g of feces and were stored at –20 °C before drying. All the fecal samples were oven-dried to a constant weight at 65 °C and were then used to analyze the DM, gross energy (GE), crude protein (CP), ether extract (EE), neutral detergent fiber (NDF), acid detergent fiber (ADF), crude fiber (CF), ash, calcium and phosphorus according to the methods described in AOAC (2007) [23]. All the indicators were also measured in feed samples. The gross energy in feces and diets was measured using an adiabatic oxygen bomb calorimeter (PARR 6400, Parr Instruments Company, Moline, IL, USA). The acid-insoluble ash in feed and feces was analyzed according to reference [24] and was set as an in vivo marker to calculate the apparent total tract digestibility (ATTD) of nutrients and energy with the following formula:

(1) $$\mathrm{Digestibility}(\%)=100-\frac{\mathrm{Indicator}\;\mathrm{in}\;\mathrm{feed}\left(\%\right)}{\mathrm{Indicator}\;\mathrm{in}\;\mathrm{feces}\left(\%\right)}\times \frac{\mathrm{Components}\;\mathrm{content}\;\mathrm{in}\;\mathrm{feces}\left(\%\right)}{\mathrm{Components}\;\mathrm{content}\;\mathrm{in}\;\mathrm{feed}\left(\%\right)}\times 100\%$$

#### 2.3.6. Lactation Energy Intake and Lactation Efficiency

The GE and digestible energy (DE) intake of sows were calculated from daily feed intake, GE in the diet and energy digestibility. The lactation efficiency of individual sows was calculated according to the method described by Rooney et al. (2020) [25]. Briefly, lactation efficiency (g of litter weight gain/MJ DE ingested by the sow) reflects the feed efficiency of lactating sows. In other words, the total energy intake of a sow was the energy intake from the feed plus the absolute value of the energy originating from maternal tissue mobilization (12.5 MJ DE per kg sow body weight), or the energy intake from feed minus the absolute value of energy used for maternal tissue deposition (20.8 MJ DE per kg of sow body weight).

### 2.4. Statistical Analysis

All data were analyzed using the PROC MIXED procedure of SAS (SAS 9.4) in a complete randomized design using the following models:

Y_ij_ = µ+ α_i_ + e_ij_

Y_ijk_ = µ+ α_i_ + β_j_ + α_i_ × β_j_ + e_ijk_

where Y (Y_ij_, Y_ijk_) is an observed trait, µ is the population mean, α_i_ is the fixed effect of the treatment (i = CON, HQ, or F + E), β_j_ is the fixed effect of a week or day (j = 0, 1, 2, or 3; 7, 14, or 21), α_i_ × β_j_ is the interaction between diet and a week/day and e (e_ij_, e_ijk_) is the residual, which was assumed to be normally distributed and to have variance homogeneity. The sow or litter was the experimental unit (*n* = 10). Model 1 was used for part of the performance of sows and ATTD, and model 2 was used to account for traits with repeated measurements (piglet performance, milk yield, milk composition and daily output, serum biochemical indexes and serum and milk supernatant antioxidant capacity). The final variance and covariance structure was chosen according to the lowest value obtained for Akaike’s Information Criterion and Bayesian information criterion in model 2. The multiple treatment comparisons were analyzed by the adjusted Tukey test. The results were shown as LSMEANS with pooled standard error. *p* < 0.05 declared a significant difference, and 0.05 ≤ *p* < 0.10 declared a tendency.

## 3. Results

### 3.1. Performance of Sows and Piglets and Predicted Milk Yield

No difference (*p* > 0.05) was observed for sow performance among the treatments (Table 2). As shown in Table 3, there was no significant difference in estimated weekly milk yield among the treatments (*p* > 0.05). Notably, sows fed with the HQ and F + E diets had higher piglet live weight (*p* < 0.05) and average daily weight gain (ADG, *p* < 0.10), litter weight gain (*p* < 0.10) and piglet growth to milk yield ratio (*p* < 0.10) as compared with the CON group. Additionally, this study shows that the weekly milk yield of sows, live weight and ADG of piglets and litter weight gain were increased (*p* < 0.05) week by week.

### 3.2. Apparent Total Tract Digestibility, Sow Energy Intake and Lactation Efficiency

The ATTD of DM, EE, GE, CP, ADF, CF and phosphorus in sows fed with the HQ diet was higher (*p* < 0.05; Table 4) as compared with the CON and F + E groups. Moreover, both the HQ and F + E groups had higher ATTD of ash (*p* < 0.05) and NDF (*p* < 0.10) than the CON group. The ATTD of phosphorus was higher in the HQ group, intermediate in the F + E group and the lowest in the CON group (*p* < 0.05). Furthermore, sows fed with the F + E diet tended to increase (*p* < 0.10) the ATTD of EE and CF as compared with the CON group. 

Compared with the HQ group, sows fed with the CON and F + E diets decreased (*p* < 0.05) the total and daily intake of DE during the lactation period. In addition, the lactation efficiency was numerically higher in both the CON and F + E groups than in the HQ group (27.33, 29.14 and 25.70 g/MJ DE, respectively; *p* = 0.28).

### 3.3. Serum Antioxidant Capacity and Biochemical Indicators

There was no significant difference in serum antioxidant capacity among treatments (*p* > 0.05; Table 5). In addition, the serum SOD and GSH-Px activities were lower (*p* < 0.05) on d 7 than on d 21, the serum CAT activity was higher (*p* < 0.05) on d 7 than on d 14 and 21 and the serum T-AOC activity was higher (*p* < 0.05) on d 7 and 21 than on d 14. 

**Figure 1:**
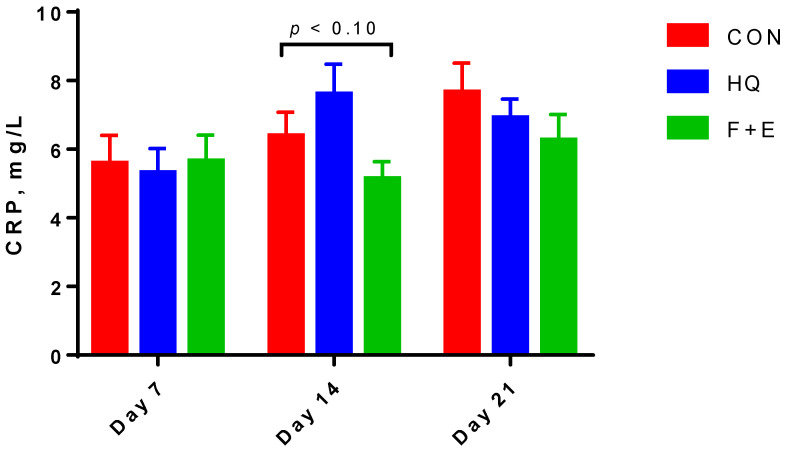
The content of serum C-reactive proteins of lactating sows. The results are presented as mean and SEM. The bars with different small letters mean a significant difference (*p* < 0.05) at the same time point, and *p* < 0.10 means a tendency. CON = control; HQ = high quality; F + E = flavor plus multi-enzymes; CRP = C-reactive protein.

The serum biochemical indicators related to protein and lipid metabolism (Table 6) show that the sows fed with the F + E diet had lower (*p* < 0.05) serum UN than the HQ and CON groups and lower serum HDL-C (*p* < 0.05) and TC (*p* < 0.10) than the HQ group. The time effect indicates that serum CREA, β-HBA, TG, NEFA, HDL-C and TC changed with the progress of lactation (*p* < 0.05). The content of CREA, β-HBA, TG and NEFA were higher (*p* < 0.05) on d 7 than on d 14 and 21, whereas the content of HDL-C and TC were lower (*p* < 0.05) on d 7 as compared with d 14 and 21. The results of serum bio-chemical indicators related to liver health and immunity indicate that the treatment by week interaction affected (*p* < 0.05) serum CRP, showing decreased (*p* < 0.10) CRP in the F + E group as compared with the HQ group on d 14 (Figure 1). In addition, the treatment effect indicates that the sows in the CON group had higher (*p* < 0.05) AST than in the F + E group. Furthermore, the lowest value of ALT was on d 14 (*p* < 0.05), and CRP was increased with the progress of lactation (*p* < 0.05).

### 3.4. Milk Composition and Daily Output

The results (Table 7) show no differences (*p* > 0.05) in milk composition among the groups. In addition, the effect of day affected milk composition and daily output. With the progress of lactation, milk DM, fat, protein and UN concentrations decreased (*p* < 0.05), whereas the lactose concentration increased (*p* < 0.05).

### 3.5. Milk Supernatant Antioxidant Capacity

**Figure 2:**
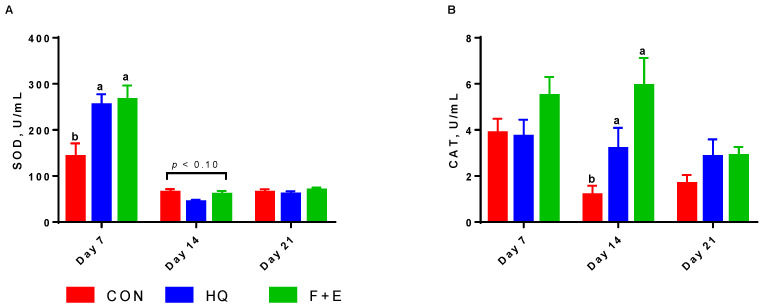
The activities of superoxide dismutase and catalase in milk supernatants of lactating sows: (**A**) The activity of SOD in milk supernatant; (**B**) The activity of CAT in milk supernatant. The results are presented as mean and SEM. The bars with different small letters mean a significant difference (*p* < 0.05) at the same time point, and *p* < 0.10 means a tendency. CON = control; HQ = high quality; F + E = flavor plus multi-enzymes; SOD = superoxide dismutase; CAT = catalase.

The results in Table 8 show that the treatment by time interactions affected (*p* < 0.05) the activities of SOD and CAT in milk supernatants (Figure 2). The CON group had the lowest (*p* < 0.05) activities of SOD on d 7 and CAT on d 14 and had an increased (*p* < 0.10) tendency of SOD activity as compared with the HQ group on d 14. There were no differences (*p* > 0.05) in milk supernatant antioxidant capacity observed between the CON and HQ groups. However, the F + E group had higher (*p* < 0.05) SOD and CAT activities as compared with the CON group, and it had higher (*p* < 0.05) GSH-Px but lower (*p* < 0.05) MDA than both the CON and HQ groups. The time effect shows that the activity of SOD and the content of MDA were higher (*p* < 0.05) on d 7 than on d 14 and 21, the CAT activity was higher (*p* < 0.05) on d 7 than on d 21 and the GSH-Px activity was higher (*p* < 0.05) on d 14 than on d 7 and 21.

## 4. Discussion

### 4.1. Performance of Sows and Their Offspring

The inclusion of flavor was reported to increase feed intake for sows’ consumed diet [8,9]; however, there was no difference in feed intake among groups in the current study. Sweeteners, such as sucrose, lactose, glucose and saccharin, are added to feed to improve the feed intake of weaned piglets or lactating sows [5,26,27,28]. Hence, the addition of sucrose (20 g/kg) and glucose (20 g/kg) in each diet in this study may explain why the palatability of feed was not improved by added flavor, which constituted glucose, aldehydes and esters as functional ingredients. The lactating sows generally became catabolic to sustain their milk production because of the insufficient intake of energy and nutrients, especially in early lactation [1,29,30,31]. Therefore, having similar feed intake resulted in no significant difference between groups in sow live weight, back fat, body composition changes and milk yield across dietary treatments. 

Milk is the major energy and nutrient source for suckling piglets before weaning [32]. In the present study, the live weight and ADG of piglets and litter weight gain were higher in the HQ and F + E groups than in the CON group, although the estimated weekly milk yield was only numerically higher in the HQ and F + E groups than in the CON group (52.90, 52.14 vs. 50.89 kg). As a result, an increased piglet growth to milk yield ratio was observed in the HQ and F + E groups, suggesting a positive effect of HQ and F + E diets on piglet performance. Furthermore, we found that the weekly milk yield of sows increased week by week, which was consistent with the results of a previous study [33], although the estimated milk yield in this study was substantially lower than that observed in high-producing hyperprolific sows [1,32].

### 4.2. Apparent Total Tract Digestibility, Energy Intake and Lactation Efficiency

Higher digestibility of feed reduces nutrient excretion through feces and provides more energy and nutrients for the animals [20]. In this study, sows in the HQ group were fed with highly digestible ingredients, such as extruded corn, extruded full-fat soybeans and fermented soybean meal, and they had higher ATTD of energy and nutrients than those in the CON group, as expected based on previous studies [34,35,36,37]. Furthermore, the addition of flavor and multi-enzymes to the CON diet improved the ATTD of EE, NDF, CF, ash and phosphorus. Likewise, previous studies indicated that dietary supplementation with multi-enzymes, including the activities of xylanase, β-glucanase and phytase, enhances nutrient digestibility [2,14,17], but little information is available on the digestibility of EE, which was also improved following multi-enzyme supplementation in the present study. The above results suggest that sows fed with the HQ and F + E diets had higher feed efficiency than the CON diet.

Increasing energy density in lactation diets has been reported to increase energy intake [25], most likely because gastric capacity is a limiting factor for feed intake during lactation. In this study, the total and daily DE intakes were higher in the HQ group compared with the CON and F + E groups. However, lactation efficiency was numerically lower in the HQ group (25.70 g/MJ), intermediate in the CON group (27.33 g/MJ) and higher in the F + E group (29.40 g/MJ). This result is consistent with the results obtained in hyperprolific sows, from which lactation efficiency decreases as dietary energy density increases [25]. The above result suggests declined growth per MJ of energy intake by a sow and lower energy efficiency from a sow to a piglet when the dietary energy density increases. 

### 4.3. Serum Antioxidation Capacity and Biochemical Indicators

In general, lactating sows experience systemic oxidative stress due to increased metabolic burden, which leads to decreased antioxidant capacity [38], especially in sows with higher body weight loss [39]. In the present study, dietary treatments had no differences in serum biochemical indicators that related to body reserve mobilization, such as CREA, β-HBA, TG and NEFA. As a result, the activities of antioxidative enzymes in the serum were only increased with the progress of lactation, but there were no differences among treatments. Previous studies indicated that the dietary inclusion of xylanase and β-glucanase improves the digestibility of amino acids in growing pigs and lactating sows [40,41,42,43], which is accompanied by decreased serum UN content [43]. Similarly, sows fed with the F + E diet decreased serum UN, indicating improved utilization of amino acids and less oxidation of proteins. Previous studies reported that higher feed intake increases TC and HDL-C levels in pigs’ serum [44], and higher energy and fat intake during the evening is accompanied by higher TC and HDL-C levels after overnight fasting in humans [45]. Hence, higher TC and HDL-C levels in sows fed with the HQ diet in the present study may be ascribed to numerically higher feed intake and significantly increased energy intake as compared with the F + E group. Sows in the F + E group had lower AST than those in the CON group, and the treatment by time interaction showed that the F + E group had lower CRP than those in the HQ group on d 14. It is known that AST is one of the biomarkers of liver damage and liver disease [46,47]. Furthermore, CRP is an acute-phase protein that participates in inflammatory processes to combat infections [48,49], and it also increases with the progress of lactation in sows [3]. Hence, although the values of biochemical indicators were in the physiological range for the species, our observations suggest that the sows fed with the F + E diet had more healthy livers and less inflammation than those in other dietary groups.

### 4.4. Milk Composition and Milk Supernatant Antioxidant Capacity

Besides milk yield, milk quality is another key factor that impacts piglets’ growth. In the current study, both HQ and F + E groups had no difference in milk composition, but there were differences in milk supernatant antioxidant capacity. A higher concentration of antioxidants is supposed to give better defense against reactive oxygen species to mammary glands and infants [50]. Previous studies showed that cows with inflammation in the udder tend to have higher MDA content and lower antioxidative enzymatic activities in milk [51,52]. In this study, we found that milk supernatant antioxidative enzymatic activities, such as SOD, CAT and GSH-Px, increased, and the content of MDA, which is a lipid peroxidation product, decreased in the F + E group compared with the other two groups. The above results indicate an elevated milk antioxidative capacity in response to the F + E diet, which may ensure healthier mammary glands and greater transfer of antioxidants to their offspring [53], thus benefiting the health of both sows and piglets. Hence, we assumed that the increased milk antioxidant capacity in the F + E group most likely reduced the maintenance energy of piglets and subsequently partitioned any milk energy into piglet growth.

## 5. Conclusions

The strategies to enhance dietary energy and nutrient concentrations or dietary ingestion and digestion using flavor and multi-enzymes improved the progeny growth of primiparous sows, but they failed to improve the feed intake of sows during the lactation period. The enhanced dietary energy and nutrients mainly acted through improving the feed digestibility and energy intake of sows, and the dietary inclusion of flavor and multi-enzymes increased the feed digestibility, antioxidant capacity and health of sows. Based on the findings in this study, it can be concluded that both a high-quality diet and the dietary supplementation of flavor and multi-enzymes in lactating sows were beneficial strategies for improving piglet growth, whereas the addition of flavor and multi-enzymes may be more promising from a health and economic perspective like enhancing utilization of cereal byproducts and thus reducing expenditure of corn and soybeans.

## Figures and Tables

**Table 1:** Diet composition and nutrient levels (fed basis).

Items	CON	HQ
Ingredients, kg		
Corn	524.20	296.86
Extruded corn		200.00
Wheat bran	150.00	100.00
Defatted rice bran	65.50	46.00
Extruded full-fat soybean	-	80.00
Fermented soybean meal	-	40.00
Soybean meal	166.00	109.00
Imported fish meal	20.00	20.00
Sucrose	20.00	20.00
Glucose	20.00	20.00
Corn oil	-	25.00
L-Lysine.H_2_SO_4_ (78.8%)	3.56	3.87
L-Methionine (99%)	-	0.51
L-Threonine (98.5%)	1.21	1.48
L-Tryptophan (98%)	0.32	0.38
L-Valine (98%)	2.00	2.27
Calcium hydrophosphate	4.64	15.82
Limestone	14.18	10.41
Premix ^1^	8.39	8.39
Total	1000.00	1000.00
Chemical compositions ^2^		
Gross energy, MJ/kg	15.79	16.76
Digestible energy, MJ/kg	13.30	14.70
Metabolizable energy, MJ/kg	12.94	13.90
Net energy, MJ/kg	9.70	10.54
Crude protein, %	16.22	17.21
Ether extract, %	2.64	5.65
Standardized ileal digestible amino acids, %		
Lys, %	0.95	1.04
Met, %	0.23	0.27
Met + Cys, %	0.52	0.56
Thr, %	0.60	0.65
Try, %	0.19	0.20
Ile, %	0.54	0.58
Val, %	0.81	0.88
Total calcium, %	0.78	0.85
Total phosphorus, %	0.80	0.96
Standard total tract digestible phosphorus, %	0.32	0.48
Neutral detergent fiber, %	18.29	14.64
Acid detergent fiber, %	4.41	4.17
Crude fiber, %	3.74	3.74

CON = control; HQ = high quality. ^1^ Provided per kilogram of diet with: VA, 6000 IU; VE, 50 IU; VD3, 1200 IU; VK3, 2.4 mg; VB1, 1 mg; VB2, 3.6 mg; VB6, 1.8 mg; VB12, 0.0125 mg; biotin, 0.24 mg; folic acid, 2 mg; niacin, 25 mg; pantothenic acid,14 mg; Fe, 100 mg; Cu, 25 mg; Zn, 125 mg; Mn, 35 mg; I, 0.2 mg; and Se, 0.3 mg. Provided per ton of diet with: sodium chloride, 4000 g; preservative, 500 g; antioxidant, 200 g; probiotics, 500 g; mycotoxin adsorbent, 1000 g; and choline chloride (60%), 1000 g. ^2^ The contents of gross energy, crude protein, ether extract, total calcium, total phosphorus, neutral detergent fiber, acid detergent fiber and crude fiber were measured values, and the digestible energy was calculated based on the analyzed dietary gross energy concentration and gross energy digestibility.

**Table 2:** Sow performance during the lactation period.

Items	CON	HQ	F + E	SEM	*p*-Value ^1^
Sows					
No. of sows	10	10	10	-	-
Feed intake					
Day 2–7, kg/d	2.22	2.41	2.40	0.212	0.77
Day 8–14, kg/d	4.74	5.02	4.41	0.267	0.29
Day 15–21, kg/d	5.77	5.90	5.60	0.252	0.71
Day 2–21, kg/d	4.34	4.54	4.22	0.197	0.51
Live weight					
Day 2, kg	198	197	196	4.9	0.97
Day 21, kg	186	186	183	4.0	0.78
Loss on day 2–21, kg	12	11	14	3.1	0.83
BFT					
BFT on day 2, mm	17.5	17.8	17.7	0.98	0.97
BFT on day 21, mm	15.5	15.7	15.6	1.00	0.98
BFT loss on day 2–21, mm	2.0	2.0	2.1	0.57	0.99
Calculated body composition					
Body lipid loss, kg	5.18	5.04	5.71	1.118	0.90
Body lipid loss, %	12.99	12.97	14.26	2.689	0.93
Body protein loss, kg	1.38	1.24	1.65	0.525	0.85
Body protein loss, %	4.32	3.91	5.28	1.785	0.86
Body energy loss, MJ	248	238	277	53.5	0.87
Body energy loss, %	10.30	10.24	11.55	2.184	0.89

CON = control; HQ = high quality; F + E = flavor plus multi-enzymes; BFT = backfat thickness. ^1^ Declare significant differences among treatments (*p* < 0.05).

**Table 3:** Piglet performance and predicted milk yield.

Items	Treatments			SEM	Time (Week)			SEM	*p*-Value ^1^		
	CON	HQ	F + E		1	2	3		Treatment	Time	Treatment × Time
Litter size, pig	8.9	9.0	8.9	0.05	9.0	8.9	8.9	0.05	0.37	0.37	0.84
Piglet live weight, kg/pig	3.55 ^b^	3.77 ^a^	3.78 ^a^	0.096	2.82 ^c^	4.32 ^b^	5.83 ^a^	0.096	0.04	<0.001	0.88
Piglet ADG, g/d	184	206	206	7.8	166^b^	214 ^a^	216 ^a^	7.8	0.07	<0.001	0.93
Litter weight gain, kg/d	1.64	1.86	1.82	0.072	1.54 ^b^	2.01 ^a^	1.92 ^a^	0.072	0.08	<0.001	0.95
Weekly milk yield, kg/week	50.89	52.90	52.14	1.062	36.64 ^c^	57.78 ^b^	61.52 ^a^	0.652	0.41	<0.001	0.67
Piglet growth:milk yield, kg/kg	0.21	0.24	0.24	0.008	0.24	0.23	0.22	0.008	0.07	0.05	0.84

CON = control; HQ = high quality; F + E = flavor plus multi-enzymes; ADG = average daily weight gain. ^1^ Declare significant differences among treatments or different time points (*p* < 0.05).

**Table 4:** Apparent total tract digestibility, sow energy intake and lactation efficiency of lactating sows.

Items	CON	HQ	F + E	SEM	*p*-Value ^1^
ATTD, %					
DM	89.40 ^b^	91.53 ^a^	89.63 ^b^	0.486	0.01
EE	64.22 ^b^	83.86 ^a^	69.44 ^b^	1.908	<0.001
GE	83.88 ^b^	87.72 ^a^	84.64 ^b^	0.575	<0.01
CP	85.18 ^b^	89.00 ^a^	86.61 ^b^	0.743	<0.01
Ash	42.10 ^b^	54.35 ^a^	50.33 ^a^	1.712	<0.001
NDF	57.41	62.49	61.86	1.703	0.08
ADF	42.69 ^b^	53.24 ^a^	40.23 ^b^	2.389	<0.01
CF	35.47 ^b^	52.55 ^a^	42.39 ^b^	2.621	<0.01
Calcium	44.68	53.38	53.60	3.451	0.134
Phosphorus	51.69 ^c^	73.25 ^a^	60.63 ^b^	2.415	<0.001
DE ^2^, MJ/kg	13.32	14.70	13.28	-	-
Lactation energy intake, MJ					
Total DE	1157.61 ^b^	1335.82 ^a^	1121.10 ^b^	53.360	0.02
Daily DE	57.88 ^b^	66.79 ^a^	56.05 ^b^	2.668	0.02
Lactation efficiency, g/MJ DE	27.33	25.7	29.14	1.517	0.28

CON = control; HQ = high quality; F + E = flavor plus multi-enzymes; ATTD = apparent total tract digestibility; DM = dry matter; EE = ether extract; GE = gross energy; CP = crude protein; NDF = neutral detergent fiber; ADF = acid detergent fiber; CF = crude fiber; DE = digestible energy. ^1^ Different superscript letters within a row declare significant differences among treatments (*p* < 0.05); ^2^ The DE was calculated based on the analyzed dietary GE concentration and GE digestibility.

**Table 5:** Serum antioxidant capacity of lactating sows.

Items	Treatments			SEM	Time (Day)			SEM	*p*-Value ^1^		
	CON	HQ	F + E		7	14	21		Treatment	Time	Treatment × Time
SOD U/mL	50.04	46.72	49.23	3.532	44.50 ^b^	46.92 ^ab^	54.56 ^a^	1.843	0.79	0.01	0.70
CAT, U/mL	12.14	10.14	11.33	0.653	15.01 ^a^	8.96 ^b^	9.65 ^b^	0.653	0.10	<0.001	0.70
GSH-Px, U/mL	1091	1088	1102	50.4	992 ^b^	1173 ^a^	1118 ^a^	38.2	0.98	0.04	0.36
T-AOC, mmol/L	0.24	0.28	0.24	0.025	0.33 ^a^	0.14 ^b^	0.28 ^a^	0.028	0.41	<0.001	0.99
MDA, nmol/mL	3.12	2.69	2.69	0.314	3.06	2.84	2.60	0.258	0.54	0.37	0.89

CON = control; HQ = high quality; F + E = flavor plus multi-enzymes; SOD = superoxide dismutase; CAT = catalase; GSH-Px = glutathione peroxidase; T-AOC = total antioxidant capacity; MDA = malondialdehyde. ^1^ Different superscript letters within a row declare significant differences among treatments or different time points (*p* < 0.05).

**Table 6:** Serum biochemical indices related to protein and lipid metabolism, liver health and immunity of lactating sows.

Items	Treatments			SEM	Time (Day)			SEM	*p*-Value ^1^		
	CON	HQ	F + E		7	14	21		Treatment	Time	Treatment × Time
Protein and lipid metabolism											
CREA, μmol/L	143.32	143.74	146.25	5.662	154.75 ^a^	138.35 ^b^	140.21 ^b^	3.954	0.93	<0.001	0.75
UN, mmol/L	8.55 ^a^	8.68 ^a^	7.27 ^b^	0.362	8.66	7.94	7.90	0.356	0.02	0.36	0.52
β-HBA, mmol/L	0.026	0.019	0.017	0.0051	0.043 ^a^	0.009 ^b^	0.010 ^b^	0.0039	0.41	<0.001	0.54
TG, mmol/L	0.47	0.43	0.45	0.023	0.57 ^a^	0.40 ^b^	0.39 ^b^	0.021	0.46	<0.001	0.54
NEFA, mmol/L	0.72	0.62	0.69	0.102	1.33 ^a^	0.33 ^b^	0.38 ^b^	0.094	0.81	<0.001	0.26
LDL-C, mmol/L	0.84	0.90	0.79	0.054	0.78	0.88	0.87	0.041	0.35	0.09	0.59
HDL-C, mmol/L	0.65 ^ab^	0.76 ^a^	0.60 ^b^	0.034	0.54 ^b^	0.68 ^a^	0.79 ^a^	0.034	<0.01	<0.001	0.39
TC, mmol/L	1.85	2.17	1.73	0.098	1.62 ^b^	1.99 ^a^	2.14 ^a^	0.098	0.07	<0.001	0.21
Liver health and immunity											
AST, U/L	34.63 ^a^	30.07 ^ab^	29.60 ^b^	1.442	33.77	29.5	31.03	1.442	0.03	0.11	0.83
ALT, U/L	27.8	25.83	25.8	1.579	26.27 ^a^	24.67 ^b^	28.50 ^a^	1.122	0.60	<0.01	0.22
GGT, U/L	35.07	38.4	38.97	2.922	38.13	37.23	37.07	1.938	0.60	0.79	0.26
CRP, mg/L	6.55	6.61	5.68	0.601	5.52 ^b^	6.38 ^ab^	6.94 ^a^	0.426	0.49	0.02	0.03

CON = control; HQ = high quality; F + E = flavor plus multi-enzymes; CREA = creatinine; UN = urea nitrogen; β-HBA = β-hydroxybutyric acid; TG = triglyceride; NEFA = non-esterified fatty acids; LDL-C = low-density lipoprotein cholesterol; HDL-C = high-density lipoprotein cholesterol; TC = total cholesterol; AST = aspartate aminotransferase; ALT = alanine aminotransferase; GGT = gamma-glutamyl transferase; CRP = C-reactive protein. ^1^ Different superscript letters within a row declare significant differences among treatments or different time points (*p* < 0.05).

**Table 7:** Milk composition and daily output of lactating sows.

Items	Treatments			SEM	Time (Day)			SEM	*p*-Value ^1^		
	CON	HQ	F + E		7	14	21		Treatment	Time	Treatment × Time
Milk composition											
DM, %	20.7	20.8	21.0	0.52	24.1 ^a^	19.1 ^b^	19.4 ^b^	0.58	0.90	<0.001	0.43
Fat, %	8.6	8.5	8.9	0.47	11.5 ^a^	7.2 ^b^	7.3 ^b^	0.47	0.72	<0.001	0.71
Protein, %	5.5	5.4	5.5	0.14	6.1 ^a^	5.1 ^b^	5.2 ^b^	0.14	0.82	<0.001	0.46
Non-fat milk solids, %	12.1	12.4	12.0	0.19	12.5	11.9	12.1	0.19	0.47	0.11	0.35
Lactose, %	5.4	5.7	5.3	0.16	5.1 ^b^	5.6 ^a^	5.7 ^a^	0.14	0.21	<0.01	0.58
SCC, ×1000 cells/mL	1614	793	1493	846.4	1998	734	1168	708.6	0.33	0.53	0.45
UN, mg/dL	52.7	52.3	51.8	2.18	60.9 ^a^	43.6 ^c^	52.3 ^b^	2.18	0.95	<0.001	0.47
Daily output											
Fat, g/d	676	686	719	40.7	822 ^a^	630 ^b^	628 ^b^	40.7	0.73	0.001	0.81
Protein, g/d	429	439	450	15.1	433	438	446	15.1	0.63	0.85	0.91
Lactose, g/d	427	472	442	15.5	366 ^b^	483 ^a^	492 ^a^	15.5	0.12	<0.001	0.85

CON = control; HQ = high quality; F + E = flavor plus multi-enzymes; DM = dry matter; SCC = somatic cell count; UN = urea nitrogen. ^1^ Different superscript letters within a row declare significant differences among treatments or different time points (*p* < 0.05).

**Table 8:** Milk supernatant antioxidant capacity of lactating sows.

Items	Treatments			SEM	Time (Day)			SEM	*p*-Value ^1^		
	CON	HQ	F + E		7	14	21		Treatment	Time	Treatment × Time
SOD, U/mL	90.51 ^b^	119.73 ^ab^	132.03 ^a^	10.283	221.01 ^a^	56.26 ^b^	64.99 ^b^	15.898	0.02	<0.001	<0.01
CAT, U/mL	2.24 ^b^	3.26 ^ab^	4.72 ^a^	0.46	4.31 ^a^	3.44 ^ab^	2.47 ^b^	0.41	<0.01	<0.01	0.08
GSH-Px, U/mL	30.54 ^b^	33.88 ^b^	44.60 ^a^	3.05	45.43 ^a^	26.88 ^b^	36.71 ^a^	3.21	<0.01	<0.01	0.65
T-AOC, mmol/L	0.89	0.98	0.74	0.099	0.89	0.84	0.88	0.09	0.25	0.88	0.52
MDA, nmol/mL	3.59 ^a^	3.53 ^a^	2.73 ^b^	0.198	4.39 ^a^	3.21 ^b^	2.25 ^c^	0.23	<0.01	<0.001	0.53

CON = control; HQ = high quality; F + E = flavor plus multi-enzymes; SOD = superoxide dismutase; CAT = catalase; GSH-Px = glutathione peroxidase; T-AOC = total antioxidant capacity; MDA = malondialdehyde. ^1^ Different superscript letters within a row declare significant differences among treatments or different time points (*p* < 0.05).

## Data Availability

Data is available upon request from the corresponding authors.

## References

[1] Strathe A.V., Bruun T.S., Hansen C.F. (2017). Sows with high milk production had both a high feed intake and high body mobilization. Animal.

[2] Cozannet P., Lawlor P.G., Leterme P., Devillard E., Geraert P.A., Rouffineau F., Preynat A. (2018). Reducing BW loss during lactation in sows: A meta-analysis on the use of a nonstarch polysaccharide-hydrolyzing enzyme supplement. J. Anim. Sci..

[3] Rosenbaum S., Ringseis R., Hillen S., Becker S., Erhardt G., Reiner G., Eder K. (2012). Genome-wide transcript profiling indicates induction of energy-generating pathways and an adaptive immune response in the liver of sows during lactation. Comp. Biochem. Physiol. Part D Genom. Proteom..

[4] Figueroa J., Solà-Oriol D., Guzmán-Pino S., Borda E., Pérez J.F. (2012). Flavor preferences conditioned by postingestive effect of sucrose and porcine digestive peptides in postweaning pigs. J. Anim. Sci..

[5] Zhang W., He H., Gong L., Lai W., Dong B., Zhang L. (2019). Effects of sweetener sucralose on diet preference, growth performance and hematological and biochemical parameters of weaned piglets. Asian Aust. J. Anim. Sci..

[6] Wang J., Yang M., Xu S., Lin Y., Che L., Fang Z., Wu D. (2014). Comparative effects of sodium butyrate and flavors on feed intake of lactating sows and growth performance of piglets. Anim. Sci. J..

[7] He L., Zang J., Liu P., Fan P., Song P., Chen J., Ma Y., Ding W., Ma X. (2017). Supplementation of milky flavors improves the reproductive performance and gut function using sow model. Protein Pept. Lett..

[8] Silva B.A.N., Tolentino R.L.S., Eskinazi S., Jacob D.V., Raidan F.S.S., Albuquerque T.V., Oliverira N.C., Araujo G.G.A., Silva K.F., Alcici P.F. (2018). Evaluation of feed flavor supplementation on the performance of lactating high-prolific sows in a tropical humid climate. Anim. Feed Sci. Technol..

[9] Wang R., Cinar M., Macun H.C., Ozenc E., Salar S. (2021). Flavor supplementation during late gestation and lactation periods increases the reproductive performance and alters fecal microbiota of the sows. Anim. Nutr..

[10] Bach Knudsen K.E. (2014). Fiber and nonstarch polysaccharide content and variation in common crops used in broiler diets. Poult. Sci..

[11] Bach Knudsen K.E., Jørgensen H., Lindberg J., Ogle B. (2001). Intestinal degradation of dietary carbohydrates–from birth to maturity. Digestive Physiology of Pigs.

[12] Kim J.C., Simmins P.H., Mullan B.P., Pluske J.R. (2005). The digestible energy value of wheat for pigs, with special reference to the post-weaned animal. Anim. Feed Sci. Technol..

[13] Le Gall M., Serena A., Jorgensen H., Theil P.K., Bach Knudsen K.E. (2009). The role of whole-wheat grain and wheat and rye ingredients on the digestion and fermentation processes in the gut-a model experiment with pigs. Br. J. Nutr..

[14] Clarke L.C., Sweeney T., Curley E., Gath V., Duffy S.K., Vigors S., Rajauria G., O’Doherty J.V. (2018). Effect of β-glucanase and β-xylanase enzymes supplemented barley diets on nutrient digestibility, growth performance and expression of intestinal nutrient transporter genes in finisher pigs. Anim. Feed Sci. Technol..

[15] Zhang H., Li Z., Tian Y., Song Z., Ai L. (2019). Interaction between barley β-glucan and corn starch and its effects on the in vitro digestion of starch. Int. J. Biol. Macromol..

[16] Walsh M.C., Geraert P.A., Maillard R., Kluess J., Lawlor P.G. (2012). The effect of a non-starch polysaccharide-hydrolysing enzyme (Rovabio^®^ Excel) on feed intake and body condition of sows during lactation and on progeny growth performance. Animal.

[17] Cozannet P., Kidd M.T., Yacoubi N., Geraert P.-A., Preynat A. (2019). Dietary energy and amino acid enhancement from a multi-enzyme preparation. J. Appl. Poult. Res..

[18] Zeng Z.K., Li Q.Y., Tian Q.Y., Xu Y.T., Piao X.S. (2018). The combination of carbohydrases and phytase to improve nutritional value and non-starch polysaccharides degradation for growing pigs fed diets with or without wheat bran. Anim. Feed Sci. Technol..

[19] Petry A.L., Patience J.F. (2020). Xylanase supplementation in corn-based swine diets: A review with emphasis on potential mechanisms of action. J. Anim. Sci..

[20] NRC (2012). Nutrient Requirements of Swine.

[21] Dourmad J.Y., Étienne M., Valancogne A., Dubois S., van Milgen J., Noblet J. (2008). InraPorc: A model and decision support tool for the nutrition of sows. Anim. Feed Sci. Technol..

[22] Hansen A.V., Strathe A.B., Kebreab E., France J., Theil P.K. (2012). Predicting milk yield and composition in lactating sows: A Bayesian approach. J. Anim. Sci..

[23] AOAC (2007). Official Methods of Analysis.

[24] Brestenský M., Nitrayová S., Heger J., Patráš P. (2017). Chromic oxide and acid-insoluble ash as markers in digestibility studies with growing pigs and sows. J. Anim. Physiol. Anim. Nutr..

[25] Rooney H.B., O'Driscoll K., O'Doherty J.V., Lawlor P.G. (2020). Effect of increasing dietary energy density during late gestation and lactation on sow performance, piglet vitality, and lifetime growth of offspring. J. Anim. Sci..

[26] Aldinger S.M., Speer V.C., Hays V.W., Catron D.V. (1959). Effect of saccharin on consumption of starter rations by baby pigs. J. Anim. Sci..

[27] Park M.S., Yang Y.X., Shinde P.L., Choi J.Y., Jo J.K., Kim J.S., Lohakare J.D., Yang B.K., Lee J.K., Kwon I.K. (2010). Effects of dietary glucose inclusion on reproductive performance, milk compositions and blood profiles in lactating sows. J. Anim. Physiol. Anim. Nutr..

[28] Liang X., Sang I.L., Lee I.S., Jin H.C., Kim I.H. (2017). Effects of saccharin (sweetener) supplementation on growth performance, fecal moisture and litter performance of lactating sows. Korean J. Agric. Sci..

[29] Eissen J.J., Apeldoorn E.J., Kanis E., Verstegen M.W., de Greef K.H. (2003). The importance of a high feed intake during lactation of primiparous sows nursing large litters. J. Anim. Sci..

[30] Beyer M., Jentsch W., Kuhla S., Wittenburg H., Kreienbring F., Scholze H., Rudolph P.E., Metges C.C. (2007). Effects of dietary energy intake during gestation and lactation on milk yield and composition of first, second and fourth parity sows. Arch. Anim. Nutr..

[31] Tokach M.D., Menegat M.B., Gourley K.M., Goodband R.D. (2019). Review: Nutrient requirements of the modern high-producing lactating sow, with an emphasis on amino acid requirements. Animal.

[32] Hojgaard C.K., Bruun T.S., Theil P.K. (2020). Impact of milk and nutrient intake of piglets and sow milk composition on piglet growth and body composition at weaning. J. Anim. Sci..

[33] Theil P.K., Jørgensen H., Jakobsen K. (2004). Energy and protein metabolism in lactating sows fed two levels of dietary fat. Livest. Prod. Sci..

[34] Muley N.S., van Heugten E., Moeser A.J., Rausch K.D., van Kempen T.A. (2007). Nutritional value for swine of extruded corn and corn fractions obtained after dry milling. J. Anim. Sci..

[35] Jing Y., Chi Y.J. (2013). Effects of twin-screw extrusion on soluble dietary fibre and physicochemical properties of soybean residue. Food Chem..

[36] Wang P., Fan C.G., Chang J., Yin Q.Q., Song A.D., Dang X.W., Lu F.S. (2016). Study on effects of microbial fermented soyabean meal on production performances of sows and suckling piglets and its acting mechanism. J. Anim. Feed Sci..

[37] Wang C., Lin C., Su W., Zhang Y., Wang F., Wang Y., Shi C., Lu Z. (2018). Effects of supplementing sow diets with fermented corn and soybean meal mixed feed during lactation on the performance of sows and progeny. J. Anim. Sci..

[38] Berchieri-Ronchi C.B., Kim S.W., Zhao Y., Correa C.R., Yeum K.-J., Ferreira A.L. (2011). Oxidative stress status of highly prolific sows during gestation and lactation. Animal.

[39] Hu L., Che L., Wu C., Curtasu M.V., Wu F., Fang Z., Lin Y., Xu S., Feng B., Li J. (2019). Metabolomic profiling reveals the difference on reproductive performance between high and low lactational weight loss sows. Metabolites.

[40] Barrera M., Cervantes M., Sauer W.C., Araiza A.B., Torrentera N., Cervantes M. (2004). Ileal amino acid digestibility and performance of growing pigs fed wheat-based diets supplemented with xylanase. J. Anim. Sci..

[41] de Souza A.L.P., Lindemann M.D., Cromwell G.L. (2007). Supplementation of dietary enzymes has varying effects on apparent protein and amino acid digestibility in reproducing sows. Livest. Sci..

[42] Nortey T.N., Patience J.F., Sands J.S., Trottier N.L., Zijlstra R.T. (2008). Effects of xylanase supplementation on the apparent digestibility and digestible content of energy, amino acids, phosphorus, and calcium in wheat and wheat by-products from dry milling fed to grower pigs. J. Anim. Sci..

[43] Zhang S., Song J., Deng Z., Cheng L., Tian M., Guan W. (2017). Effects of combined alpha-galactosidase and xylanase supplementation on nutrient digestibility and growth performance in growing pigs. Arch. Anim. Nutr..

[44] Rauw W.M., Portolés O., Corella D., Soler J., Reixach J., Tibau J., Prat J.M., Diaz I., Gómez-Raya L. (2007). Behaviour influences cholesterol plasma levels in a pig model. Animal.

[45] Chen H.J., Chuang S.Y., Chang H.Y., Pan W.H. (2019). Energy intake at different times of the day: Its association with elevated total and LDL cholesterol levels. Nutr. Metab. Cardiovasc. Dis..

[46] Ge Y., Zhang Q., Jiao Z., Li H., Bai G., Wang H. (2018). Adipose-derived stem cells reduce liver oxidative stress and autophagy induced by ischemia-reperfusion and hepatectomy injury in swine. Life Sci..

[47] Su R.C., Lard A., Breidenbach J.D., Kleinhenz A.L., Modyanov N., Malhotra D., Haller S.T., Kennedy D.J. (2020). Assessment of diagnostic biomarkers of liver injury in the setting of microcystin-LR (MC-LR) hepatotoxicity. Chemosphere.

[48] McKimmie R.L., Daniel K.R., Carr J.J., Bowden D.W., Freedman B.I., Register T.C., Hsu F.-C., Lohman K.K., Weinberg R.B., Wagenknecht L.E. (2008). Hepatic steatosis and subclinical cardiovascular disease in a cohort enriched for type 2 diabetes: The Diabetes Heart Study. Am. J. Gastroenterol..

[49] Sproston N.R., Ashworth J.J. (2018). Role of C-Reactive Protein at Sites of Inflammation and Infection. Front. Immunol..

[50] Lipko-Przybylska J., Kankofer M. (2012). Antioxidant defence of colostrum and milk in consecutive lactations in sows. Ir. Vet. J..

[51] Yigit A.A., Cinar M., Macun H.C., Ozenc E., Salar S. (2018). Total oxidant and antioxidant activities in milk with various somatic cell count intervals during discrete cow and buffalo lactation periods. Indian J. Dairy Sci..

[52] Zigo F., Elecko J., Vasil M., Ondrasovicova S., Farkasova Z., Malova J., Takac L., Zigova M., Bujok J., Pecka-Kielb E. (2019). The occurrence of mastitis and its effect on the milk malondialdehyde concentrations and blood enzymatic antioxidants in dairy cows. Vet. Sci..

[53] Hu H., Wang M., Zhan X., Li X., Zhao R. (2011). Effect of different selenium sources on productive performance, serum and milk Se concentrations, and antioxidant status of sows. Biol. Trace Elem. Res..

